# Electrical stimulation modulates Wnt signaling and regulates genes for the motor endplate and calcium binding in muscle of rats with spinal cord transection

**DOI:** 10.1186/1471-2202-14-81

**Published:** 2013-08-02

**Authors:** Yong Wu, Lauren Collier, Weiping Qin, Graham Creasey, William A Bauman, Jonathan Jarvis, Christopher Cardozo

**Affiliations:** 1 Center of Excellence for the Medical Consequences of SCI, James J. Peters VA Medical Center, 130 West Kingsbridge Road, Bronx, NY 10468, USA; 2 Department of Medicine, Mount Sinai School of Medicine, New York, NY, USA; 3 Department of Rehabilitation Medicine, Mount Sinai School of Medicine, New York, NY, USA; 4 VA Palo Alto Health Care System, Stanford University, Palo Alto, CA, USA; 5 School of Biomedical Sciences, University of Liverpool, Liverpool, UK

**Keywords:** Spinal cord injury, Paralysis, Electrical stimulation, Exercise, Gene expression

## Abstract

**Background:**

Spinal cord injury (SCI) results in muscle atrophy and a shift of slow oxidative to fast glycolytic fibers. Electrical stimulation (ES) at least partially restores muscle mass and fiber type distribution. The objective of this study was to was to characterize the early molecular adaptations that occur in rat soleus muscle after initiating isometric resistance exercise by ES for one hour per day for 1, 3 or 7 days when ES was begun 16 weeks after SCI. Additionally, changes in mRNA levels after ES were compared with those induced in soleus at the same time points after gastrocnemius tenotomy (GA).

**Results:**

ES increased expression of Hey1 and Pitx2 suggesting increased Notch and Wnt signaling, respectively, but did not normalize RCAN1.4, a measure of calcineurin/NFAT signaling, or PGC-1ß mRNA levels. ES increased PGC-1α expression but not that of slow myofibrillar genes. Microarray analysis showed that after ES, genes coding for calcium binding proteins and nicotinic acetylcholine receptors were increased, and the expression of genes involved in blood vessel formation and morphogenesis was altered. Of the 165 genes altered by ES only 16 were also differentially expressed after GA, of which 12 were altered in the same direction by ES and GA. In contrast to ES, GA induced expression of genes related to oxidative phosphorylation.

**Conclusions:**

Notch and Wnt signaling may be involved in ES-induced increases in the mass of paralyzed muscle. Molecular adaptations of paralyzed soleus to resistance exercise are delayed or defective compared to normally innervated muscle.

## Background

Spinal cord injury (SCI) causes substantial loss of skeletal muscle mass, endurance, fiber cross sectional area, and strength (for reviews, see [1-3]). Reduced resistance to fatigue is associated with a shift in muscle fiber type from slow to mixed or fast twitch fibers which are less able to generate ATP by oxidative phosphorylation to support repetitive contractions, and are thus more quickly fatigued [1-3]. The fiber type of an individual muscle cell is determined by its specific content of contractile isoforms and the mix of enzymes involved in ATP generation. Determinants of fiber type include signaling through calcineurin/NFAT in concert with activation of the transcriptional coregulator PGC-1α [4]. PGC-1α is also a master regulator of mitochondrial biogenesis and oxidative phosphorylation [4]. Following SCI in male rats, nuclear levels of PGC-1α are reduced, as is expression of slow-twitch fiber type genes and genes for enzymes needed for oxidation of fats and carbohydrates to generate ATP [5].

Neuromuscular activity induced by electrical stimulation (ES) of nerves is capable of reducing or reversing at least some adverse effects of SCI on muscle. In rats with spinal isolation (SI), implanted microstimulators prevent muscle loss when stimulation of muscle contraction is provided [6]. SI is a variant of SCI in which reflex arcs below the level of the spinal cord transection are disrupted through additional surgeries that include cutting thoracic and lumbar dorsal nerve roots. Similarly, ES prevents loss of muscle in individuals with SCI [7], and exercise using functional ES (FES)-induced muscle contractions increases muscle mass, force of muscle contraction, and muscle endurance and reverses at least partially slow to fast fiber type changes [8-14].

Very little is known about the molecular adaptations that underlie the effects of ES to restore a more normal function to chronically paralyzed muscle or how such adaptations compare to those of a normally innervated muscle exercised in a similar manner. Adaptation of skeletal muscle to exercise has been shown to involve molecular responses that include the activation of several fundamental signaling networks. Signaling through Notch has been implicated in muscle hypertrophy in response to testosterone or resistance exercise [15-17]. In rodents, hypertrophy in response to muscle overloading was associated with and requires increased Wnt/ß-catenin signaling [18,19]. In slow-twitch, but not fast-twitch muscle, hypertrophy has been suggested by some studies to also involve signaling through the calcium-dependent calcineurin/NFAT pathway [20,21]. Expression of PGC-1α and downstream genes for mitochondrial biogenesis is increased rapidly after exercise [22,23]. A study of gene expression changes in 2 individuals with SCI in whom ES had been used to train the soleus muscle for 6 years showed that ES increased expression of slow-twitch fiber genes and genes encoding PGC-1α and proteins involved in metabolism of carbohydrates and lipids to generate ATP [24]. While one might expect rapid increases in expression of such genes after initiating ES, in patients with SCI, 4 weeks of ES did not alter fiber types in the tibialis anterior muscle [25]; it is not clear whether this finding reflects a delay in activation of signaling by which programs for expression of such gene expression or a defect in the ability of neuromuscular activity to activate the molecular signals necessary to upregulate these genes. The possibility that there are fundamental impairments in response of paralyzed muscle to training using ES after SCI has not been systematically explored.

**Figure 1:**
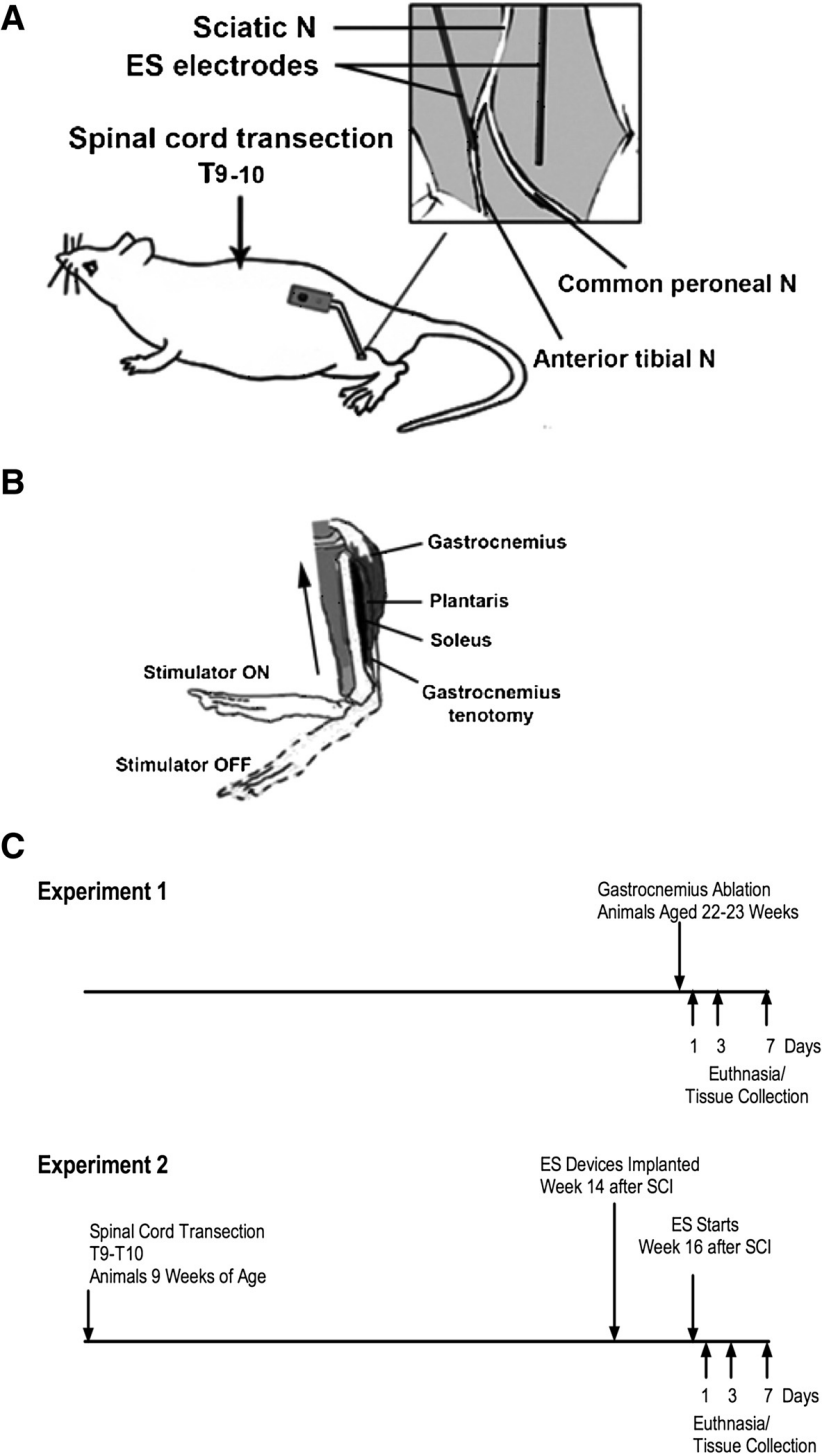
**Overview of the experimental design. A**. The placement of electrodes and the body of the stimulator are shown. **B**. Effects of stimulation on position of the foot are shown. **C**. The time-course of the studies with electrical stimulation (ES) and gastrocnemius tenotomy is shown.

The primary goal of this investigation was to gain insight into the early molecular adaptations that occur after initiating isometric resistance exercise in rats with SCI. Isometric exercise was provided by ES that was begun 16 weeks after SCI and performed daily for 1, 3 or 7 days in female rats with a complete transection of the spinal cord at T10. Properties of muscle from these animals were compared to those for animals with SCI that were not trained with ES and with animals that had sham SCI surgery. Real-time PCR was used to examine changes in expression levels of selected genes involved in Notch, Wnt and calcineurin signaling, as well as genes for PGC-1α and slow-twitch myofibrillar genes. In addition, an unbiased assessment of changes in gene expression was performed using high density oligonucleotide DNA microarrays. We used soleus muscle for these studies as it is a slow-twitch muscle that might be expected to show large alterations in these pathways, and for which effects of long-term ES on gene expression in humans have been reported [24]. To provide insight as to how exercise responses of a paralyzed muscle differ from a normal one, gene expression changes induced by ES were compared to those that occur when a normally innervated soleus is overloaded by a distal tenotomy of a synergist, the gastrocnemius (GA) [26,27]. Design of the experiments is shown in Figure 1.

## Results

### Effects of SCI, ES and GA on body and muscle weights

When compared to animals that underwent a sham SCI procedure (Sham-SCI), body weights were increased by 6.7% for the SCI group that did not receive ES (SCI) and by 8.8% for the SCI group trained with ES (SCI-ES) for 7 days (Table 1). These trends were also present for preoperative body weights and thus most likely reflect differences in the size of the animals in the cohorts used.

**Table 1:** Body and left hindlimb muscle weights

**Treatment**	**Number of animals**	**Pre Op weight (g)**	**Final weight (g)**	**Left soleus (g/g)**	**Left plantaris (g/g)**
SHAM-SCI	13	232.9 ± 9.5	238.0 ± 14.5	0.049 ± 0.005	0.100 ± 0.017
SCI	8	241.5 ± 13.7	254.3 ± 23.6^a^	0.028 ± 0.009^a^	0.057 ± 0.014^a^
1 day ES	5	231.4 ± 10.2	259.9 ± 22.1	0.035 ± 0.009^a^	0.049 ± 0.006^a^
3 Day ES	4	227 ± 7.2	255.3 ± 8.9	0.037 ± 0.005^a^	0.074 ± 0.011^a^
7 Day ES	5	240.4 ± 11.3	259.0 ± 29.6^a^	0.032 ± 0.003^a^	0.072 ± 0.012^a^
1 Day GA	8	253.8 ± 18.3	251.90 ± 20.0	0.050 ± 0.005	0.090 ± 0.012
1 Day Sham GA	7	260.1 ± 16.7	256.7 ± 16.0	0.047 ± 0.003	0.087 ± 0.013
3 Day GA	8	279.1 ± 20.7	273.3 ± 18.1	0.051 ± 0.006	0.090 ± 0.006^b^
3 Day Sham GA	8	282.5 ± 22.0	275.8 ± 21.7	0.048 ± 0.009	0.081 ± 0.005
7 Day GA	8	268.0 ± 12.5	259.0 ± 12.3	0.055 ± 0.009^b^	0.089 ± 0.015
7 Day Sham GA	8	279.5 ± 23.3	277.0 ± 19.8	0.043 ± 0.004	0.081 ± 0.005

Muscle weights are normalized relative to preoperative body weights and are shown as mean ± SEM. ^a^ p <0.05 versus Sham-SCI. ^b^ p < 0.05 versus the Sham-GA group at the same time point. *Abbreviations*: *SCI* spinal cord injured, *ES* electrical stimulation, *GA* gastrocnemius ablation, *Op* operative.

SCI reduced plantaris muscle weights by 42% and soleus muscle weights by 43% (Table 1). The weights of the plantaris and soleus muscles from the SCI-ES animals at 1, 3 or 7 days of ES were not significantly different from those for the SCI animals and remained significantly reduced compared to Sham-SCI animals (Table 1). After 7 days of ES, when effects of ES on muscle weight might be expected to be the greatest, the weights of the left and right plantaris from SCI-ES animals were 0.072 ± 0.005 and 0.059 ± 0.002; this difference was not significant (p < 0.067). The weight of the left soleus was greater than that of the right soleus for SCI-ES animals at 7 days (p < 0.0017). The increase in soleus muscle weight after 7 days of ES indicates that some muscle hypertrophy occurred with the ES paradigm used.

In the GA model, body weights were similar for Sham-GA and GA groups; two-way ANOVA for muscle weights showed a main effect of GA for plantaris (p < 0.01) and soleus (p < 0.01) but not for time. Plantaris weight was increased by 11% at 3 days after GA, and appeared to be 9.9% greater at 7 days although this change did not reach significance. At 7 days after GA, soleus muscle mass was also increased (p < 0.01) by approximately 28% compared to soleus from Sham-GA animals at the same time point.

### Genome-wide analysis of the effects of SCI on muscle gene expression

**Figure 2:**
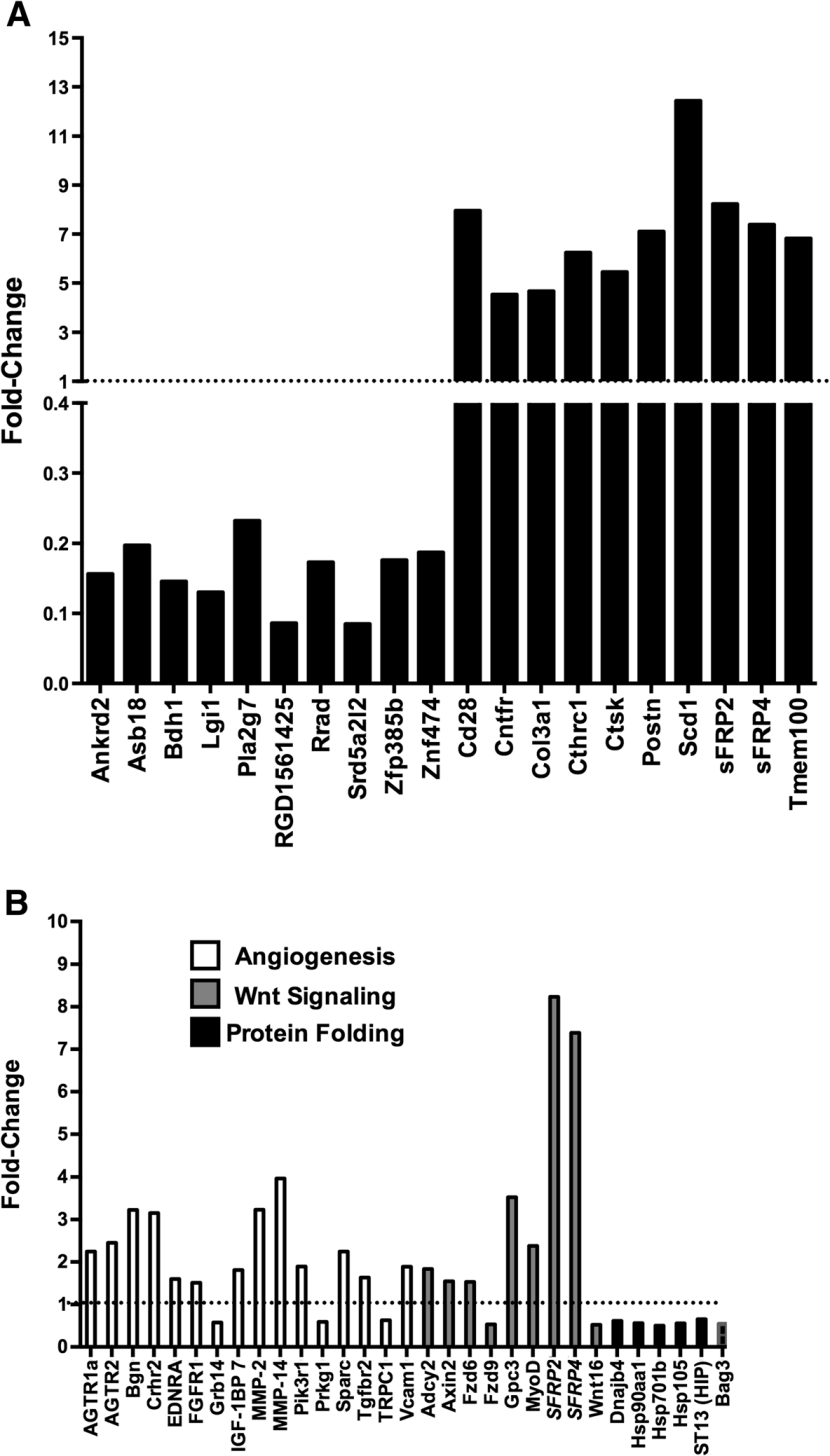
**Evaluation by microarray of the expression level of mRNAs in soleus muscle from SCI animals relative to Sham-SCI animals. A**. Expression levels for the 20 most altered genes are shown as mean fold-change. **B**. Expression values for genes represented in functionally enriched GeneGo Process Networks are shown, with the functionally enriched network to which each gene belongs designated as shown in the legend inset into panel **B**. Dotted lines in panels **A** and **B** denote the expression values in Sham-SCI animals. Expression values are mean fold-change for SCI animals relative to Sham-SCI; N = 3 animals for each group.

Microarray analysis revealed 404 genes that were altered by at least 1.5 fold in soleus at 17 weeks after SCI, of which 219 were upregulated and 185 were downregulated. Among the 10 most upregulated genes were the Wnt inhibitors sFRP2 and sFRP4, as well as periostin (Postn), which is a protein linked to angiogenesis and cell mobility, and ciliary neurotrophic factor receptor (Cnftr) (Figure 2A). Highly downregulated genes included Ankrd2 (Figure 2A), as well as the calcineurin inhibitors RCAN1 and RCAN2, the slow-twitch myosin heavy chain gene MyH7b, and the calcium binding protein calsequestrin 2 (Additional file 1: Table S1).

GeneGo analysis of genes altered by SCI was performed to identify functionally enriched biological themes represented by these genes. Among the most highly enriched themes were angiogenesis and blood vessel morphogenesis, protein folding, and Wnt signaling (Figure 2B). All genes categorized as being involved in protein folding were downregulated, and to a similar extent (Figure 2B). Most genes for Wnt signaling (7 out of 9) were upregulated with marked increases observed for sFRP2 and sFRP4 as noted above; Wnt16, was downregulated (Figure 2B). The majority of genes for angiogenesis and blood vessel morphogenesis were also upregulated (13 out of 16) (Figure 2B). Among GeneGo Pathway and Metabolic networks, glutathione and lipid metabolism were also enriched.

### ES effects on selected signaling genes in soleus muscle

**Figure 3:**
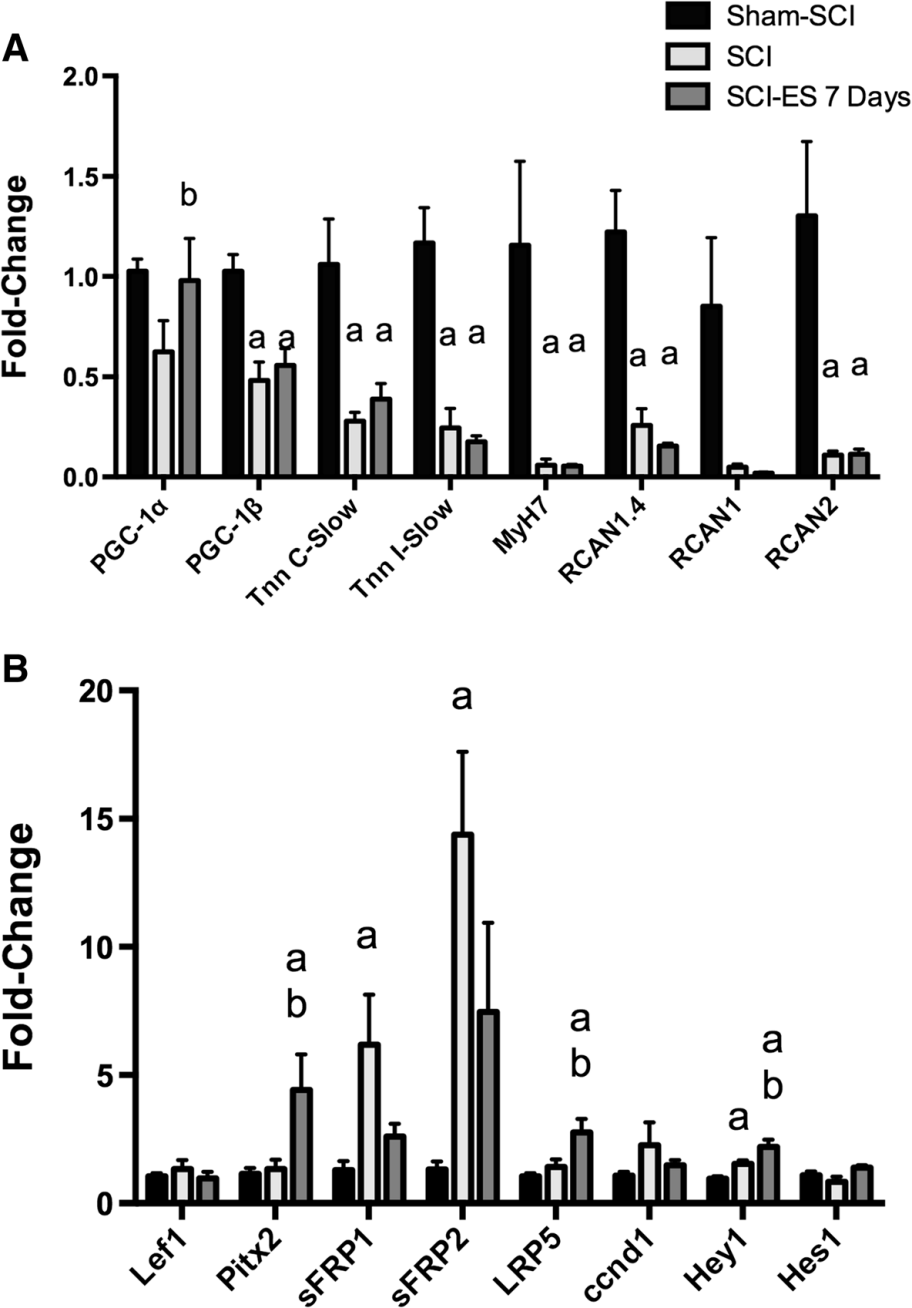
**Assessment by qPCR of the effects of ES for 7 days on mRNA levels of PGC-1α-responsive genes, and genes involved in signaling for calcineurin, Wnt/ß-catenin and Notch. A**. mRNA levels for fiber-type related genes. **B**. mRNA levels for Wnt signaling genes. mRNA levels were determined by real time PCR in samples of the left soleus from animals from the indicated groups. Data are means ± SEM where N was 13, 8 and 5 animals for Sham-SCI, SCI, and SCI-ES, respectively. Letters above each bar indicate the significance of differences as follows: a, p < 0.05 versus Sham-SCI; b, p < 0.05 versus SCI; the remaining differences did not reach statistical significance.

To learn how ES altered expression of genes that participate in programs necessary for muscle hypertrophy and for control of muscle fiber type and oxidative metabolism, the effects of ES on mRNA levels for transcripts of such genes was examined by qPCR. Stimulation of soleus by ES in humans for 6 years increased expression of PGC-1α and genes for oxidative metabolism as well as altered slow myosin heavy chain isoforms expression [24]. To evaluate early effects of ES on levels of such transcripts, mRNA levels for PGC-1α and PGC-1ß, as well as several genes typical of slow-twitch fibers, were determined by qPCR. PGC-1α mRNA levels appeared to be reduced by SCI, although not significantly, and were increased by ES (Figure 3A). PGC-1ß mRNA levels were reduced by SCI by 52% and were not increased at 7 days after initiating ES. The slow-twitch fiber genes MyH7, Tnn I-slow and Tnn C-slow were reduced by SCI by 70-95%, but these genes were not increased by ES at 7 days (Figure 3A). Expression of the calcineurin/NFAT-sensitive transcript RCAN1.4 was reduced by SCI and, unexpectedly, was not altered by ES at 7 days (Figure 3A). Expression of RCAN2 was reduced by SCI 91% and was also unaffected by ES at 7 days (Figure 3A); a similar pattern was observed for RCAN1, although these differences did not reach significance (F = 3.16, p 0.068, ANOVA).

Both Notch and Wnt signaling pathways have been implicated in muscle growth and repair during adulthood [16-18,28]. To test how 7 days of ES altered Notch and Wnt signaling after SCI, expression of several genes involved in these signaling pathways in soleus muscle was examined using qPCR. SCI did not alter expression of Pitx2 or Lef1, which are Wnt-responsive genes [29,30], but Pitx2 expression was increased by ES for 7 days (Figure 3B). Expression of the Wnt co-receptor LRP5 followed the same pattern, being unchanged after SCI, and increased at 7 days after starting ES (Figure 3B). Expression of the Wnt inhibitors sFRP1 and sFRP2 was increased after SCI and appeared to be reduced by 7 days of ES, although these changes did not reach significance (Figure 3B). Expression of the Notch target gene Hey1 was upregulated by approximately 1.5-fold after SCI, and further increased by 7 days of ES, suggesting increases in Notch signaling; expression of another Notch target gene, Hes1, was not significantly altered (Figure 3B).

### Genome-wide analysis of the effects of ES gene expression in soleus muscle

To gain further insight into early responses of soleus to ES, gene expression profiles in soleus were examined across time by microarray analysis. A total of 165 different genes were altered by at least 1.5 fold at one or more time points after initiating ES. The largest number of genes (129) was altered at 1 day, of which 90 were upregulated and 39 were downregulated. At both 3 and 7 days after initiating ES, 29 genes were regulated. Correlation between microarray and qPCR results was calculated for the SCI and SCI-ES7 groups for RCAN1, RCAN2, and sFRP2. These mRNAs were chosen because each was significantly altered by both microarray and qPCR approaches. Expression changes showed good agreement between the two techniques (R^2^ 0.76, p < 0.025).

**Figure 4:**
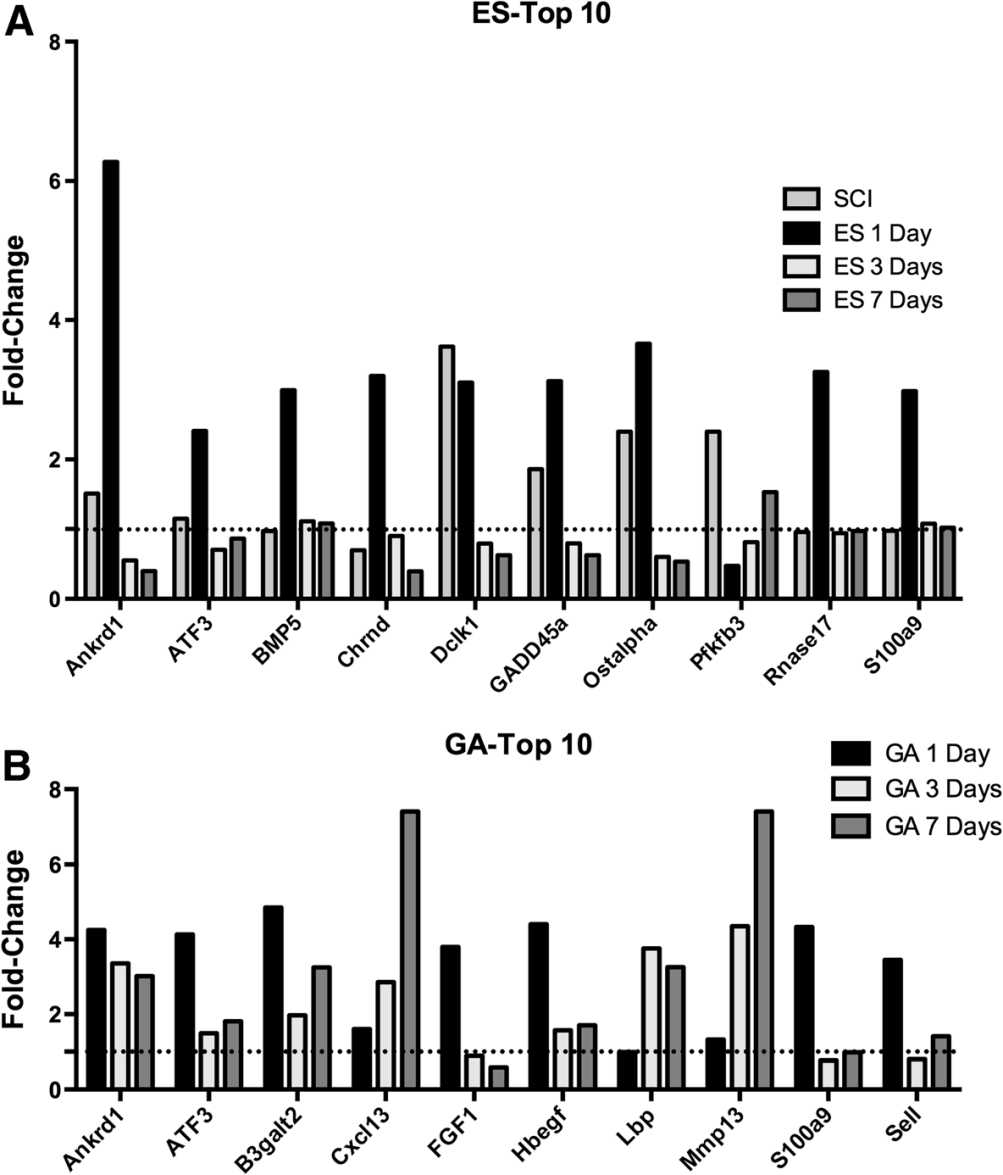
**Relative expression levels the 10 most upregulated genes identified by microarray analysis after electrical stimulation (ES) or gastrocnemius ablation (GA). A**. Mean gene expression is shown across time expressed as fold-change relative to Sham-SCI. **B**. Mean gene expression values expressed as fold-change relative to the Sham-GA group from the same time point. Dotted lines denote the expression values in Sham-SCI or Sham-GA animals, respectively. N = 3 animals per group.

Among the most altered genes was Ankrd1, which was upregulated at 1 day after starting ES (Figure 4A). Other genes upregulated at 1 day after starting ES included the nicotinic acetylcholine receptor subunit D (Chrnd), and the calcium binding protein S100a9 (Figure 4A). Several genes upregulated by SCI were downregulated after initiating ES, including Dclk1, GADD45a, and Ostalpha, a solute transporter (Figure 4A).

**Figure 5:**
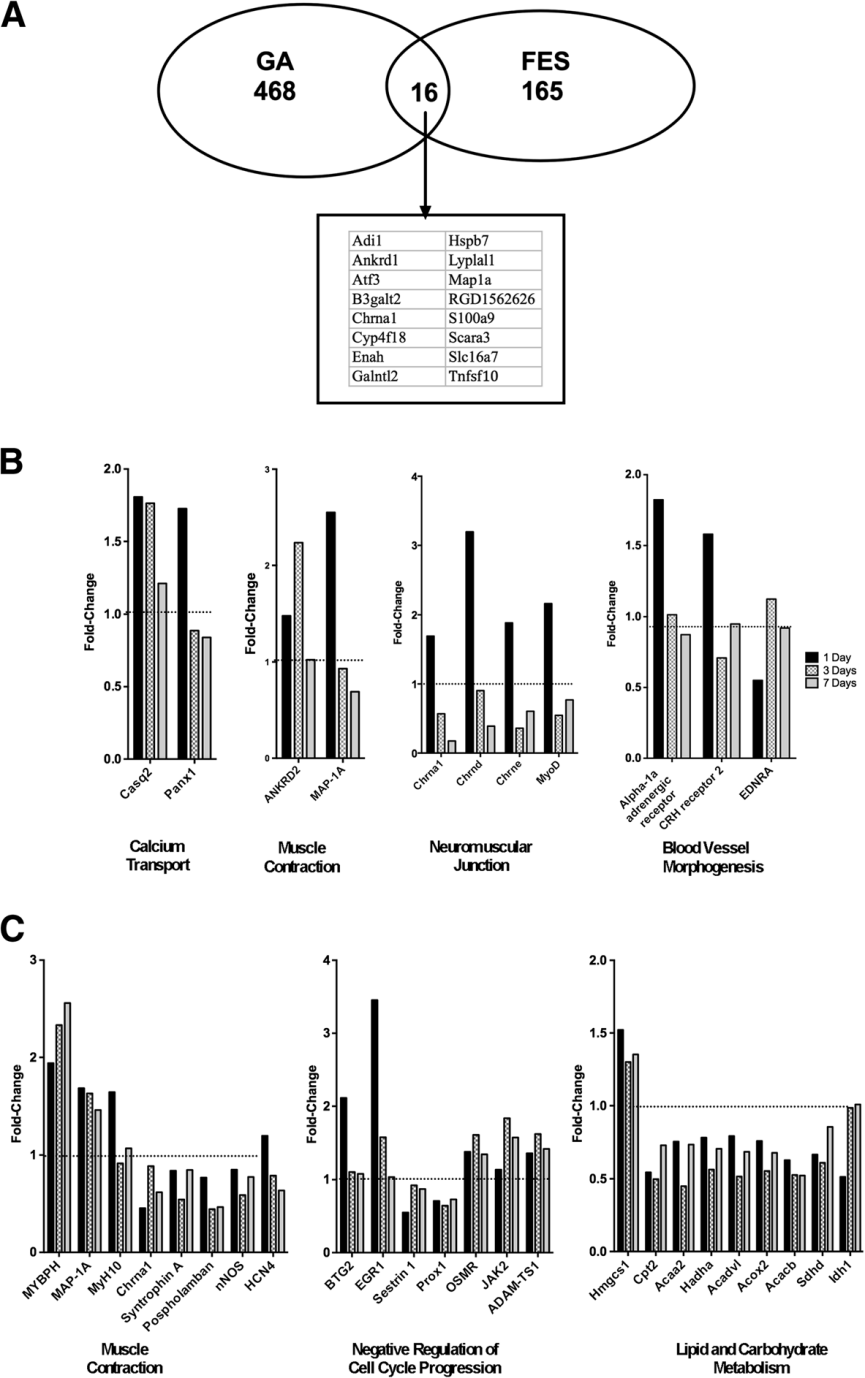
**Differences between electrical stimulation (ES) and gastrocnemius ablation (GA) in the functionally enriched categories for differentially expressed genes as determined by microarray analysis. A**. Venn diagram depicting the number of total genes altered by ES or GA, and the number of genes altered by both. **B**. Selected functional categories enriched in genes regulated after ES, and mean gene expression values for differentially expressed genes grouped in those categories, expressed as fold-change relative to Sham-SCI. **C**. Selected functionally enriched categories enriched in genes regulated by GA and mean expression values for the corresponding genes expressed as fold-change relative to values in the Sham-GA group at the same time point. Dotted lines in panels **A** and **B** denote the expression values in Sham-SCI or Sham-GA animals, respectively. N = 3 animals per group.

To gain further insights into the functional implications of the gene expression changes elicited in soleus by ES, an analysis of enrichment in biological themes represented by the genes altered in soleus by ES was performed. Pathways that were highly represented among genes regulated by ES included fatty acid omega oxidation, mitochondrial fatty acid beta-oxidation, and CoA-biosynthesis. GeneGo Process networks included muscle contraction, neuromuscular junction, blood vessel morphogenesis, and calcium transport. All together, 11 genes were included in these processes, of which 10 were upregulated at 1 day, and one was downregulated (Figure 5B). Calcium transport and muscle contraction genes were upregulated at 1 day and had returned to near normal levels by 7 days (Figure 5B); a similar pattern of altered expression was observed for the blood vessel morphogenesis genes α-1 adrenergic receptor and CRH receptor 2 (Figure 5B). Neuromuscular junction genes were upregulated at 1 day and downregulated by 7 days (Figure 5B).

### Comparison of alterations to the transcriptome in the soleus muscle over time after ES or GA

To gain insight as to how gene expression changes stimulated by ES of muscle paralyzed by SCI compared to those induced by overload of the soleus by GA, gene expression was assessed over time in soleus muscle at 1, 3 and 7 days after GA. Following GA, 468 genes were altered by at least 1.5 fold at one time point or more, with the greatest number of altered genes, 309, being observed at 3 days after GA; of these 309 genes, 140 were downregulated and 169 upregulated.

Genes regulated by GA were strikingly different from those regulated by ES. Only 16 of 468 genes regulated by GA were also regulated by ES (Figure 5A), and all but 3 of these genes were altered at 1 day after initiating these muscle overload paradigms. In addition, fewer genes were altered by ES than GA at every time point. Overall, ES altered approximately 65% fewer genes. Of the 10 genes most highly upregulated by GA, only 3 were among the 10 most altered genes by ES, specifically Ankrd1, ATF3 and s100a9 (Figure 4A and 4B). Similar changes in expression level and temporal profile were observed for S100a9 and ATF3 for GA and ES, with an increase at 1 day and subsequent return toward baseline expression values at 3 and 7 days (Figure 4A and 4B). By contrast, after GA, Ankrd1 levels were increased at all times (Figure 4B), whereas Ankrd1 levels increased at 1 day after ES then declined (Figure 4A).

Biological processes and pathways represented by differentially expressed genes were also compared for ES and GA. After GA, GeneGo Process networks that were most greatly enriched were related to muscle contraction, negative regulation of cell cycle progression, and lipid and carbohydrate metabolism (Figure 5C), a result quite different from the list of enriched pathways observed after ES (Figure 5B). After GA, genes related to oxidative reduction represented another enriched functional category, which included genes such as PGC-1α.

## Discussion

### Gene expression changes after complete spinal cord transection

In female rats at 17 weeks after SCI there were was enrichment in genes involved in angiogenesis and blood vessel morphogenesis, protein folding and Wnt signaling. Alterations in genes involved in the formation of blood vessels might be expected given the decrease in skeletal muscle mass and presumed remodeling of capillary beds that must also occur. Blood flow to the lower extremities is reduced after SCI reflecting diminished skeletal muscle mass [2]. Capillary density of the tibialis anterior after SCI is similar to or somewhat less than that for able-bodied controls [25], indicating that after SCI substantial remodeling of capillary networks must have occured during atrophy after SCI. The decrease in expression of genes supporting normal protein folding might also be explained as a reflection of an atrophying muscle that requires less de-novo synthesis of proteins.

More surprising was the enrichment in genes for Wnt signaling, particularly the marked upregulation of the Wnt inhibitors sFRP1, sFRP2 and sFRP4. The elevations in expression of Wnt inhibitors must be interpreted together with changes of Pitx2, a Wnt responsive gene [29]. That Pitx2 expression was unaltered at 17 weeks after SCI may indicate that Wnt signaling overall was unchanged by SCI. One must also consider that Wnts function primarily as autocrine and paracrine regulators and that inhibition of Wnt signaling in specific microenvironments, such as the satellite cell niche, may not be reflected by Pitx2 levels in whole muscle. Although decreased Wnt signaling has not been shown to accelerate disuse atrophy of muscle, one might suggest that diminished Wnt signaling is detrimental to maintaining muscle mass, because Wnt signaling has been implicated in muscle hypertrophy. Specifically, ß-catenin, through which canonical Wnt signaling regulates gene expression, has been found in a transgenic mouse model to be necessary for overload-induced muscle hypertrophy [18,19]. Findings that signaling by Wnt7a through Fzd7 activates Akt/mTOR signaling [28] suggests there is also ß-catenin independent Wnt signaling that underlies the anabolic activity of Wnts. Wnt signaling is also critical to fate commitment and differentiation of myogenic precursors, and essential for the repair of injured skeletal muscle in adulthood [31,32], and one may view the hypertrophic response of atrophied muscle as a reparative process following SCI.

Calcineurin activity has been shown to be exquisitely sensitive to neuromuscular activity [20,33]. As such, it was not surprising that levels of the NFAT-sensitive transcript RCAN1.4 were greatly reduced after SCI. An unexpected finding was that expression of the calcineurin inhibitors RCAN1 and RCAN2 was also diminished after SCI. One interpretation of this result is that expression of these genes is regulated in part by calcineurin in an inhibitory feedback loop, such that expression levels of RCAN1 and RCAN2 fall when calcineurin activity is low. In contrast to these findings in muscle after SCI, RCAN2 expression rises by 35 days after sciatic nerve transection in gastrocnemius muscle [34]. Why RCAN2 expression differs in these two model systems is not clear, although gender may be one factor. Another may be a difference in spontaneous action potentials in motor neurons, which are electrically silent after nerve transection, whereas there is some evidence that motor neurons are periodically activated after SCI, presumably through spinal reflex arcs [35].

PGC-1α mRNA was not reduced at 17 weeks after SCI, while PGC-1ß was reduced; marked reductions in mRNA levels for MyH7, Tnn C-slow and Tnn I-slow were also observed. Consistent with these findings, in a previous report examining changes in gastrocnemius muscle at 56 days after SCI, decreases were observed for PGC-1ß protein, and for expression of mRNA and proteins for slow myofibrillar components [5]. However, in contrast to the current study, mRNA levels for PGC-1α were reduced in gastrocnemius muscle by greater than 50% at 56 days after SCI in male rats associated with a similar reduction in PGC-1α protein in whole muscle and nearly complete loss of PGC-1α from nuclear fractions obtained by subcellular fractionation [5]. Differences in PGC-1α mRNA levels between these two studies may relate to differences in muscle studied, because levels of PGC-1α are elevated in slow twitch muscle such as soleus compared to fast twitch muscles [36]. Reasons for the differences in effects of SCI on PGC-1α levels after SCI between males and females could also be gender-related. In male rats, administration of a replacement dose of testosterone combined with nandrolone markedly increased PGC-1α mRNA and protein in muscle of SCI rats [5], indicating that PGC-1α is an androgen responsive gene.

### Effects of ES on gene expression in soleus muscle

The current study showed that over the first 7 days after initiating ES in a female rat model of SCI, alterations in soleus gene expression were notable for genes were involved in the Notch and Wnt signaling pathways, neuromuscular junctions, vascular remodeling, and calcium transport, and for a lack of change in PGC-1α or of slow myofibrillar proteins. Several studies of the effects of ES on gene expression of individuals with SCI have been conducted. Twenty four hours after resistance exercise in humans with SCI, increased levels for IGFBP4, MyoD, myogenin, p21-Waf1, and IGF-1 were observed [37,38]. In separate studies of individuals with SCI, prolonged periods of exercise training using ES promoted gains in skeletal muscle mass and strength that were associated with increased expression of citrate synthase, GLUT1 and GLUT4 [39]; improved insulin sensitivity was reported in one study [39] and a trend toward improvements in insulin responsiveness was found in a second [40].

The finding in the present study that ES did not alter expression of slow myofibrillar proteins in soleus was unexpected because in normally innervated muscle, neuromuscular activity rapidly increases expression levels of genes for mitochondrial proteins [23], and because in humans, long-term studies of ES have shown increased expression of slow-twitch fiber genes and genes encoding proteins involved in metabolism of carbohydrates and lipids to generate ATP [24]. Expression of slow fiber genes is upregulated by calcineurin/NFAT signaling and PGC-1α [4] but neither PGC-1α expression nor calcineurin/NFAT signaling assessed by RCAN1.4 levels appeared to have changed after ES, suggesting one explanation for the lack of effect of ES on such genes. Our data do not permit one to exclude the possibility that RCAN1.4 mRNA has a short half-life, which could preclude detection of transient rises in levels of this transcript at the time tissues were harvested, which was approximately 24 hours after the last session of ES. Evidence of a delay in fiber type changes after initiating ES has also been reported in humans in whom 4 weeks of ES did not alter fiber types in the tibialis anterior muscle [25]. Why upregulation of slow myofibrillar genes is delayed when muscle is exercised after SCI by ES is unclear. One possibility is that during the early period after initiating ES, ES injured muscle fibers, as discussed further below. Such injury might limit the ability of muscle fibers to respond appropriately to increased neuromuscular activity. It is also possible that one or more factors necessary for full activity of PGC-1α and/or calcineurin/NFAT in upregulating such genes is absent early after initiating ES, or not fully activated by 7 days of ES.

Recent observations that activation of mTOR, a requisite step for muscle hypertrophy, may not involve growth factor/PI3 kinase/Akt signaling [41-43] has led to the exciting question of what other signals might stimulate overload-induced hypertrophy. In this regard, a novel aspect of the above findings was that in the soleus muscle of rats with SCI, expression of both Hey1 and Pitx2 was elevated after 7 days of ES, suggesting that there were increases in both Notch and Wnt/ß-catenin signaling. Signaling through Notch and Wnt is necessary for muscle repair after injury [31,32], and has been implicated in signaling for muscle hypertrophy in some experimental models but not others. Increased expression of Notch has been implicated in muscle hypertrophy resulting from testosterone in mice and humans [16,17]. Expression of a Notch-responsive gene, Hey2, was found to be increased in female rats at 4 and 24 hours after a bout of resistance exercise but was unaltered in male rats [15]. As noted above, activation of ß-catenin and upregulation of Wnt signaling genes has been found to be associated with overload-induced muscle hypertrophy [18,28], although specifically how Wnt signals to stimulate hypertrophy remains unknown.

A quite unexpected effect of ES was the upregulation of genes encoding proteins that form the neuromuscular junction, specifically subunits of the nicotinic acetylcholine receptor (e.g., Chrnd). One interpretation of this finding is that by 17 weeks after SCI, an insufficient number or density of such receptors is present at motor endplates to support normal neuromuscular transmission, the failure of which is one potential mechanism for reduced contractile response to neural inputs. Skeletal muscle membrane potential has been reported to be reduced over the first 30 days after spinal cord transection in rats, associated with diffusion of nicotinic acetylcholine receptors away from motor endplates [44]. Alternative explanations should certainly be considered and may include aborization of axons and subsequent formation of additional neuromuscular junctions.

Influx of calcium across the cytoplasmic membrane and release of calcium from the sarcoplasmic reticulum and subsequent reuptake of calcium are critical to excitation-contraction coupling. Failure of excitation-contraction coupling is believed to be an important mechanism underlying the easy fatigability observed after SCI [45]. Information as to how calcium storage and transport is altered in muscle after SCI is limited. In one report, SCI was found to induce a mismatch between the slow and fast isoforms of the calcium transporter SERCA and fiber type [46]. It is thus notable that calcium transport was also an enriched GeneGo theme over time in soleus muscle after ES. Among genes related to calcium binding and transport were those of calsequestin 2 and S100a9. Calsequestin is thought to be the major protein responsible for storing calcium within the lumen of the sarcoplasmic reticulum [47,48], and release of calcium from calsequestin results in increased cytoplasmic calcium levels, thereby initiating contraction. Two isoforms of calsequestin have been identified, with calsequestin 2 found in slow-twitch muscle and heart. Upregulation of calsequestin 2 in response to ES might improve the capacity of muscle for excitation-contraction coupling by increasing the reservoir of calcium binding sites within the SR.

### Comparison of gene expression changes in the soleus muscle after ES or GA

The comparison of gene expression changes elicited in soleus muscle by ES and GA revealed a small number of genes regulated by both forms of exercise, but overall showed very different profiles, and marked differences in the functional categories for which differentially expressed genes were over-represented. Notably under-represented among the genes regulated by ES were those associated with slow-twitch fibers, metabolism of substrates in energy metabolism and oxidative phosphorylation. One might infer that the genes regulated by both ES and GA play critical roles in adaptation of skeletal muscle to resistance exercise, and that the overall poor overlap in genes differentially expressed after ES or GA reflects early differences in the ability of muscle to adapt to resistance exercise, or in the hitherto unexplored mechanisms by which such adaptation occurs in the two paradigms.

The absence of changes after ES in PGC-1α, slow-fiber genes, and many PGC-1α target genes of energy metabolism suggest impairments or delays in the response of paralyzed and atrophied muscle to neuromuscular activity elicited in our study by ES. When considering this interpretation of the data it should be noted that the isometric exercise achieved in our study by ES for 1 hour each day is of shorter duration than GA, which overloads soleus during every periods of activity; in addition, GA produces non-isometric overloading of the soleus. However, other reports have also found impaired exercise responses of such genes in skeletal muscle after SCI, supporting the view that differences observed between ES and GA were, at least in part, attributable to underlying properties of muscle rather than the nature of the exercise. Studies in man suggest that changes in expression of genes for slow-twitch fibers and energy metabolism do not occur at 4 weeks after initiating ES [25]. Importantly, longer periods of exercise by ES stimulate increases in slow oxidative type I and fast oxidative type IIA fibers, increased capacity for oxidative phosphorylation, and increased contractile force associated with diminished fatigability [1-3]. Why there is a delay in upregulation of such genes occur is unknown.

Several reports suggest that during periods of disuse, the normal protective mechanisms that minimize tissue injury from the mechanical or metabolic demands of exercise are diminished or lost. A single bout of resistance exercise in persons with SCI produced greater muscle injury than a similar exercise in controls [49]; similarly, studies of muscle from rats ambulating after periods of muscle disuse due to spaceflight or hindlimb suspension have shown increased myofiber damage and inflammation [50]. The identity of the genes involved in altering gene expression programs to protect skeletal muscle against the demands of exercise are not well understood but are likely to include PGC-1α and its downstream target genes. Loss of PGC-1α from skeletal muscle reduces exercise tolerance [51], and PGC-1α has been reported to favorably alter levels of antioxidants, and thus to reduce oxidant stress [51,52].

Genes altered by both ES and GA included two with reported roles in muscle biology: Ankrd1 and ATF3. ATF3 is a transcription factor upregulated by cellular stress [53] with an uncertain role in adaptation of muscle to inactivity or exercise; it has been associated with pathological cardiac hypertrophy and dysfunction [54]. Ankrd1 is one of 3 muscle ankyrin repeat domain proteins (MARPs) and appears to function in part at least by dissociating from its typical binding sites at the I band to undergo nuclear translocation and participate in transcriptional regulation thereby serving as a link between the contractile apparatus and nucleus [55]. Ankrd1 does not appear to be essential to normal function, because mice lacking Ankrd1 appear to have a normal phenotype, but mice lacking all MARPs have subtle abnormalities in muscle contractile properties and stiffness [56]. Whether Ankrd1 is needed for proper response to muscle overloading, or optimal recovery of muscle after periods of inactivity, is unknown. A role for Ankrd1 in muscle pathology is suggested by findings that dilated cardiomyopathies may be linked to Ankrd1 mutations [57,58].

## Conclusions

Our findings suggest that during initial adaptations to ES after 4 months of disuse due to complete spinal cord transection, muscle must repair or regenerate vasculature and cellular elements and structures, including neuromuscular junctions and calcium storage machinery of the sarcoplasmic reticulum. Wnt signaling appears to be involved in initial responses of muscle to ES, consistent with the role of this pathway in hypertrophy of normally innervated muscle [18,28]. A novel finding from this study is upregulation of Hey1 by ES, which suggests a role for Notch in the hypertrophy of atrophied muscle in response to exercise. The large difference in gene expression profiles observed between ES and GA may reflect a delay in the ability of muscle to respond to ES as a result of relatively prolonged disuse and associated atrophy. It should be noted that the muscle atrophy after SCI is a form of disuse atrophy and that findings from this study may be pertinent to exercise responses of skeletal muscle atrophied as a consequence of other conditions such as bed rest, immobilization from a cast, or spaceflight. After longer periods of ES, many expected adaptations to exercise, such as increased PGC-1α and proteins for oxidative phosphorylation, have been observed in soleus from two subjects with SCI. Why such changes are delayed after initiating ES is an interesting question for future studies.

## Methods

### Animals

All studies and procedures with experimental animals were approved by the Institutional Animal Care and Use Committee at the James J. Peters Veterans Affairs Medical Center. All studies were conducted in conformance with the recommendations of the NIH Guide for the Care and Use of Laboratory Animals. All surgeries were conducted under anesthesia with inhaled isofluorane. Every effort was made to minimize the suffering of animal subjects. Female Wistar rats were obtained from Taconic Farms (Germantown, NY) and housed in temperature and humidity-controlled rooms with a 12:12 hour day:night cycle. Animals were provided water and standard rat chow ad libitum. Two experiments were performed as outlined below.

### Experiment 1: effects of gastrocnemius ablation (GA)

Rats between 22 and 23 weeks of age were anesthetized by inhalation of isofluorane. Hair on the lower left hindlimb was removed with a clipper and skin was cleaned with alcohol and betadine. The distal insertion of the gastrocnemius muscle into the Achilles tendon was separated from the remainder of the tendon by blunt dissection and cut. Skin was closed with suture. A second group of animals received a sham ablation that was identical except that the insertion of the gastrocnemius into the Achilles tendon was not cut. At 1, 3 and 7 days after surgery, animals were weighed and anesthetized with isofluorane prior to the removal of soleus and plantaris muscles by careful dissection. Muscles were weighed then snap-frozen by immersion in liquid nitrogen. Animals were euthanized by aortic transection while anesthetized by inhalation of isofluorane.

### Experiment 2: effects of ES on muscle gene expression

#### ***Characteristics of the ES system employed***

An ES paradigm was developed wherein near-isometric co-contraction of soleus, plantaris and tibialis anterior was stimulated by electrodes placed adjacent to the anterior tibial and common peroneal nerves (Figure 1A and 1B). In preliminary experiments, cutting the distal insertion of the gastrocnemius prevented concurrent contraction of this muscle from overwhelming the opposing force of the contracting tibialis anterior. Preliminary studies established that at low stimulation amplitudes, the ankle was extended, and that as stimulation voltage increased, ankle flexion occurred until the ankle was at approximately 90 degrees. Further ankle flexion was not achieved with increases in stimulation voltage. Preliminary studies showed that stimulation parameters of 1.5 volts at 40 Hz consistently and reproducibly elicited near-isometric co-contraction of soleus, tibialis anterior and plantaris. These stimulation parameters were used for experiments.

Electrical stimulation for these studies was accomplished using single channel implantable electrical stimulators consisting of a microcontroller-based circuit and battery encapsulated in silicone rubber. The devices used were described previously [59]; a slightly larger battery was used in this application to provide a longer lifetime. Stimulators were equipped with a light sensor by which stimulation patterns and stimulation amplitude could be selected using light passing through the skin provided by a coded series of flashes from a stroboscope triggered by a microprocessor-controlled programming device. A light-emitting diode in the microstimulator provided a series of flashes visible through the skin immediately after a programming sequence by which it could be confirmed that the commands were correctly interpreted by the device.

#### ***Experimental design and SCI surgery***

The experiment included 3 groups of animals: Sham-SCI, SCI, and SCI-ES where ES was provided for 1, 3 or 7 days. At 9 weeks of age SCI and SCI-ES animals underwent a complete spinal cord transection by the following procedures. Animals were anesthetized by inhalation of isofluorane and hair was removed with a clipper. Skin over the back was cleaned with betadine and isopropyl alcohol. After making a midline incision (2 cm) centered over the interspace between the 9^th^ and 10^th^ vertebral bodies, the spinal cord was visualized by removing the vertebral processes of the 9^th^ and 10^th^ vertebrae with a bone rongeur, and the spinal cord was transected with microscissors. The space between transected ends of the spinal cord was filled with surgical sponge and the wound was closed in 2 layers with suture. Urine was expressed 3 times daily until automaticity developed, then as needed. Baytril was administered for the first 3 to 5 days postoperatively then as indicated for cloudy or bloody urine or for overt wound infection. Sham-SCI animals received an identical surgery, including a laminectomy, except that the spinal cord was not cut.

#### ***Implantation of ES stimulators***

Stimulators were implanted 14 weeks after the spinal cord transection. Animals were anesthetized by inhalation of isofluorane. The left hind limb and hip were shaved and the underlying skin was cleaned using betadine and isopropyl alcohol. A small incision was made over the left hip parallel to the femur. Using blunt dissection, the division of the left sciatic nerve was exposed and a pocket was created under the skin over the lower back for the stimulator. The stimulator was inserted into the pocket and sutured in place. One electrode was placed such that it nearly touched the anterior tibial nerve about 8 mm distal to the division of the sciatic nerve. The second was placed about 2 mm away from the common peroneal nerve and 1 cm distal to the trifurcation of the sciatic nerve. A tenotomy of the distal insertion of the gastrocnemius into the Achilles tendon was then performed following the procedures outlined above. SCI animals underwent all surgeries for implantation of the stimulators, and left-sided gastrocnemius tenotomy, but did not have stimulators implanted.

#### ***ES stimulation and tissue collection***

Beginning at 16 weeks after SCI, that is, 2 weeks after implanting stimulators in the ES group, ES was initiated for 1 hour per day using stimulation at 1.5 volts and 40 Hz. Each ES cycle consisted of 2 second periods of stimulation followed by 18 seconds rest. The ES groups were euthanized at 1, 3 or 7 days. The SCI animals were euthanized 21 days after the Sham-implantation surgery. Tissues were excised, weighed and stored as described above for the GA studies. An outline of the time course of procedures employed is provided (Figure 1). Tissue collection and euthanasia were performed as described above for Experiment 1.

### Microarray analysis

Soleus muscle was selected for microarray expression analysis because it is the major slow-twitch muscle in the lower hindlimb, and response of soleus to isometric contraction after SCI has been studied in significant detail in man [24]. Total RNA was isolated from soleus muscles using Trizol reagent then further purified using RNAeasy minicolumns (Qiagen). Analysis of RNA using an Agilent Bioanalyzer revealed that integrity of RNA was greater than 8 for all samples. Microarray analysis was performed using Affymetrix rat exon 1.0 ST arrays following the manufacturer’s recommended procedures and was performed by the Microarray Core Facility at the Children’s National Medical Center. Baseline subtraction, quantile normalization and median polishing of expression values for transcripts were performed using the Oligo package [60] for Bioconductor [61]. Data were then annotated and filtered in MeV 4.8 [62] to exclude probes with intensities less than 3.0, or variance across all arrays less than 50% (e.g., probes for which there was little difference in expression level across groups). Genes with significantly different expression levels across Sham-SCI and SCI groups were identified by one-way ANOVA. Genes significantly different between the Sham-GA and and GA groups were identified by two-way ANOVA. For each time point these lists of differentially expressed genes were then filtered to exclude transcripts that were unassigned to genes and to identify genes altered by at least 1.5 fold for that time point (1, 3 or 7 days of ES or GA); the same filter was applied for the comparison of SCI and Sham-SCI groups. These filtered lists of genes were tested for enrichment in biological themes using GeneGo by Metacore.

### Real time PCR (qPCR)

Total RNA isolated as above was subjected to on the column digestion with DNAase I, eluted, and analyzed for RNA integrity using an Agilent Bioanalyzer 2100. RNA integrity values were greater than 8 for all samples. cDNA libraries were synthesized using the High Capacity cDNA Reverse Transcription Kit (Applied Biosystems). Briefly, real time PCR was performed using an Applied Biosystems Via 7 thermocycler, and Invitrogen Taqman 2X PCR Master Mix. Invitrogen Assays on Demand probes were used for all assays except for RCAN1.4 (previously called MCIP1.4), for which an assay was designed by Applied Biosystems using mRNA sequence data downloaded from the internet. The assay was based on the fact that RCAN1.4 is unique in containing exon 4, and can be detected in qPCR assays for the boundary between exons 4 and 5. Samples were assayed in triplicate, and means of triplicates were used in subsequent calculations. Changes in mRNA levels were calculated using the 2^-∆∆Ct^ method [63], and 18S RNA was used to normalize expression values. The control group used to calculate fold-change for the SCI-ES groups was Sham-SCI. For the GA groups, the Sham-GA group at the same time point was used as the control.

### Statistics

The significance of differences among means for SCI, SCI-ES and Sham-SCI animals was tested by one-way ANOVA with a Newman-Keuls test post-hoc. The significance of differences across time and between GA and Sham-GA was tested using two-way ANOVA with a Holm-Sidak’s test post hoc. A paired students t-test was used to compare muscle weights for the left and right hindlimb. Significance was set at p < 0.05.

### Supporting data

The data sets supporting the results of this article are available in the NIH GEO repository under accession number GSE37476 at http://www.ncbi.nlm.nih.gov/geo/query/acc.cgi?targ=self&form=html&view=quick&acc=GSE37476.

## Abbreviations

ES: Electrical stimulation; FES: Functional electrical stimulation; GA: Gastrocnemius distal tenotomy; SCI: Spinal cord injury.

## Competing interests

The authors declare that they have no competing interests.

## Authors’ contributions

Yong Wu extracted RNA and conducted the qPCR analysis; Lauren Collier conducted the animal studies; Weiping Qin was involved in study design and data interpretation; Graham Creasey helped with study conceptualization and organization and edited the manuscript; William Bauman helped with data interpretation and edited the manuscript; Jonathan Jarvis developed the ES concept, designed and built the ES stimulators and control systems and edited the manuscript; Christopher Cardozo conceived, organized and oversaw the implementation of the project, led data analysis, and wrote the manuscript. All authors read and approved the final manuscript.

## Supplementary Material

Additional file 1: Table S1 Listing of significant genes when comparing expression for SCI and Sham-SCI groups where Group means are Log-base 2 of expression values for 3 animals per group. **Table S2.** Listing of significant genes altered by at least 1.5 fold when comparing SCI-ES 1 Day and SCI-Sham-ES where Group means are Log-base 2 of expression values. **Table S3.** Listing of significant genes altered by at least 1.5 fold when comparing SCI-ES 3 Days and SCI-Sham-ES where Group means are Log-base 2 of expression values. **Table S4.** Listing of significant genes altered by at least 1.5 fold when comparing SCI-ES 7 Days and SCI-Sham-ES where Group means are Log-base 2 of expression values. **Table S5.** Listing of significant genes altered by at least 1.5 fold when comparing OL-1 Day and Sham-OL 1 Day where Group means are Log-base 2 of expression values. **Table S6.** Listing of significant genes altered by at least 1.5 fold when comparing OL-3 Days and Sham-OL 3 Days where Group means are Log-base 2 of expression values. **Table S7.** Listing of significant genes altered by at least 1.5 fold when comparing OL-7 Days and Sham-OL 7 Days where Group means are Log-base 2 of expression values. Click here for file
